# Impact of malaria during pregnancy on pregnancy outcomes in a Ugandan prospective cohort with intensive malaria screening and prompt treatment

**DOI:** 10.1186/1475-2875-12-139

**Published:** 2013-04-24

**Authors:** Pierre De Beaudrap, Eleanor Turyakira, Lisa J White, Carolyn Nabasumba, Benon Tumwebaze, Atis Muehlenbachs, Philippe J Guérin, Yap Boum, Rose McGready, Patrice Piola

**Affiliations:** 1 Epicentre, Paris, France; 2 UMI 233, Institut de Recherche pour le Développement, Université Montpellier I, Montpellier, France; 3 Epicentre, Mbarara, Uganda; 4 Mbarara University of Science and Technology (MUST), Mbarara, Uganda; 5 Mahidol-Oxford Tropical Medicine Research Unit, Faculty of Tropical Medicine, Mahidol University, Bangkok, Thailand; 6 Centre for Tropical Medicine, Nuffield Department of Medicine, University of Oxford, Oxford, UK; 7 University of Washington, Seattle, WA, USA; 8 WorldWide Antimalarial Resistance Network (WWARN), Oxford, United Kingdom; 9 Shoklo Malaria Research Unit (SMRU), Mahidol University, Mae Sot, Thailand; 10 Institut Pasteur, Antananarivo, Madagascar

**Keywords:** Malaria in pregnancy, Birth outcomes, Sub-Saharan Africa, Cohort

## Abstract

**Background:**

Malaria in pregnancy (MiP) is a major public health problem in endemic areas of sub-Saharan Africa and has important consequences on birth outcome. Because MiP is a complex phenomenon and malaria epidemiology is rapidly changing, additional evidence is still required to understand how best to control malaria. This study followed a prospective cohort of pregnant women who had access to intensive malaria screening and prompt treatment to identify factors associated with increased risk of MiP and to analyse how various characteristics of MiP affect delivery outcomes.

**Methods:**

Between October 2006 and May 2009, 1,218 pregnant women were enrolled in a prospective cohort. After an initial assessment, they were screened weekly for malaria. At delivery, blood smears were obtained from the mother, placenta, cord and newborn. Multivariate analyses were performed to analyse the association between mothers’ characteristics and malaria risk, as well as between MiP and birth outcome, length and weight at birth. This study is a secondary analysis of a trial registered with ClinicalTrials.gov, number NCT00495508.

**Results:**

Overall, 288/1,069 (27%) mothers had 345 peripheral malaria infections. The risk of peripheral malaria was higher in mothers who were younger, infected with HIV, had less education, lived in rural areas or reported no bed net use, whereas the risk of placental infection was associated with more frequent malaria infections and with infection during late pregnancy. The risk of pre-term delivery and of miscarriage was increased in mothers infected with HIV, living in rural areas and with MiP occurring within two weeks of delivery.

In adjusted analysis, birth weight but not length was reduced in babies of mothers exposed to MiP (−60g, 95%CI: -120 to 0 for at least one infection and -150 g, 95%CI: -280 to −20 for >1 infections).

**Conclusions:**

In this study, the timing, parasitaemia level and number of peripherally-detected malaria infections, but not the presence of fever, were associated with adverse birth outcomes. Hence, prompt malaria detection and treatment should be offered to pregnant women regardless of symptoms or other preventive measures used during pregnancy, and with increased focus on mothers living in remote areas.

## Background

Despite numerous studies conducted over the last decades, malaria in pregnancy (MiP) remains an important public health problem that has proved difficult to tackle. Many studies from areas with different malaria transmission patterns have investigated the consequences of MiP on both maternal health and birth outcomes. While the consequences of MiP on maternal health are dominated by anaemia, data on malaria-related maternal mortality are sparse [1]. For the foetus, the most commonly reported adverse effect of MiP is an increased risk of low birth weight (LBW) [2-5], which, in turn, is a significant risk factor for both impaired development [6-8] and infant mortality [9,10]. However, most of these studies used only a single measurement point (from cross-sectional surveys or at delivery) to identify MiP and, therefore, do not capture the multiple factors that play a role over an extended period of time.

While reliable assessment of MiP is critical to elucidating its impact on birth outcomes and infant health, it is problematic because many factors (some of which are difficult to fully capture) are relevant to a complete understanding. MiP may be either continuous or intermittent, depending on a woman’s exposure to vectors, level of immunity and possible co-infections (e.g. other malaria species, HIV or helminths), and on the efficacy of treatment and prevention interventions available to her. Tools to measure parasite presence are limited by their sensitivity and by how often women attend antenatal care services; hence MiP is often only partially observed. To more fully evaluate the impact of MiP on both maternal and infant outcomes, investigations must consider multiple aspects of malaria infection, such as timing, frequency, intensity and severity of the infections, as well as the treatment provided.

Recent studies focused on one or a few features of malaria, such as timing and/or frequency [11-15], or the effect of a single infection early in pregnancy (when weekly screening was routinely provided throughout pregnancy [13]), and have produced inconsistent results. Several investigations found that LBW risk was associated specifically with malaria infections occurring in early pregnancy [11,14,15]. In contrast, a study conducted in Benin reported a higher risk of LBW associated with malaria infection after six months of pregnancy [12], and data from Thailand did not show a significantly lower birth weight in newborns of mothers with a single treated malaria episode in the first trimester compared to newborns of mothers without malaria infection [13]. Likewise, conflicting results have also been reported on the association between the number of malaria infections and the risk of LBW [11,14-16].

MiP is thought to affect birth outcomes through two mechanisms, intrauterine growth restriction (IUGR) and preterm delivery, which might - at least partially - explain these discordant findings. It has been estimated that MiP in settings with stable malaria transmission in Africa is potentially responsible for up to 70% of IUGR and 36% of preterm delivery [4]. The former has been consistently associated with placental infection [17-24], while the latter appears to correlate with systemic manifestations of malaria infection in the mother [25-27]. However, accurate determination of gestational age is required to distinguish IUGR from preterm delivery—a determination that is difficult to make in resource-constrained settings, where tools such as ultrasound are rarely available. As a result, evidence of the relative importance of IUGR versus preterm delivery due to MiP remains limited [28].

In recent years control of MiP has relied partly on intermittent preventive treatment (IPT), with WHO currently recommending at least two doses with sulphadoxine-pyrimethamine (SP) [29]. However, growing resistance of malaria parasites to SP in many regions [30,31], combined with the changing epidemiology of malaria, indicate that other prevention approaches must be strengthened. To help fill the evidence gap regarding the impact of MiP on delivery outcomes in accurately dated pregnancies, this study reports on the findings from a prospective cohort of pregnant women with access to weekly antenatal malaria screening and prompt treatment.

## Methods

### Population and setting

The study was conducted in Mbarara district, southwestern Uganda. This predominantly rural area lies at an altitude of about 1,500 m above sea level and has moderate levels of malaria transmission [32]. Between October 2006 and May 2009, 1,218 pregnant women with an estimated gestational age ≥13 weeks were enrolled in a prospective observational cohort. The first 1197 women in this cohort screened for malaria with a positive rapid diagnostic test (RDT) confirmed by a positive blood smear were invited to participate in an additional study comparing the efficacy and tolerance of artemether–lumefantrine with oral quinine for the treatment of uncomplicated falciparum malaria published elsewhere [33].

### Clinical and monitoring procedures

At baseline, a comprehensive assessment of the pregnant women’s socio-demographic characteristics and health status was performed, including a medical and obstetrical history, clinical and obstetric examination, ultrasound evaluation, blood smear and hemoglobin measurement. Estimated gestational age by was determined by ultrasound in all women enrolled in the study between week 16–20 of pregnancy (72% of the cohort). For the remaining mothers, i.e., those recruited after the 20^th^ week of gestation, we turned to a published model that predicts gestational age from symphysis-fundal height (SFH) measurements and calibrated it using the data from the 16–20 week group [34], and then used these results to predict gestational age at delivery in the subset of mothers without ultrasound (Additional file 1).

After this initial assessment, the mothers returned to the clinic weekly for a clinical examination and malaria RDT. In case of positive RDT, malarial infection was confirmed with a blood smear. Treatment of uncomplicated falciparum malaria included a random allocation of artemether-lumefantrine for three days or quinine for seven days. Infections with only *Plasmodium vivax* were treated with chloroquine. All women in the cohort received standard supervised IPT with two doses of SP given at intervals of one month or more during the second and third trimesters, as well as iron and folate supplementation, antihelmintic treatment and insecticide-treated bed nets (ITN). All treatments were provided free-of-charge.

At delivery, blood smears were obtained from the mother, placenta, cord and newborn to test for the presence of *Plasmodium* and malaria pigment. Placental histology was available only for a subset of the cohort (n=260). Placental malaria cases were classified according to the presence of parasitized erythrocytes, intervillous inflammation and haemozoin deposition [18,35]. Newborns were given an initial standardized physical examination by a medical officer, weighed to the nearest 10g using a SECA mechanical type scale, and measured for length to the nearest centimeter using a portable stadiometer (Shorr productions, US). Infants delivered outside of a health facility were examined within 24 hours of birth by a study medical officer.

### Laboratory procedures

Paracheck® RDTs were performed using a finger-prick blood sample and interpreted according to the manufacturer’s instructions. Thick and thin blood smears were prepared and stained with Giemsa. Parasitaemia was calculated by counting parasites against 200 white blood cells (or 500, if nine parasites or fewer were counted against 200 white blood cells). Placental smears were taken by incising a fresh placenta on the maternal surface halfway between the cord and the periphery, and were then examined for the presence of parasites and pigment [35].

HIV testing and treatment was proposed to all participants and performed according to national guidelines [36], which include cotrimoxazole prophylaxis for people infected with HIV. Haemoglobin was measured from a fingerprick sample by the Haemocue B-Haemoglobin analyzer (Ängelholm, Sweden).

### Definitions

Low birth weight was defined as <2,500 g measured within 24 hours of birth; preterm as newborn gestational age <37 weeks at delivery; stillbirth as the delivery of a non-living foetus ≥28 weeks gestation; and miscarriage as the delivery of a non-viable foetus either at <28 weeks gestation or weighing <500 g.

### Statistical analysis

### Malaria infection in pregnancy model

Various parameters of malaria exposure during pregnancy were described and analysed for their temporal change and for their association with maternal characteristics or study interventions that may have affected MiP characteristics. Peripheral malaria was defined as the occurrence of a positive peripheral blood smear. After a treated malaria episode, a subsequent episode was considered a recurrence only after a minimum of 14 days, with at least one negative blood smear during this period [10]. Placental malaria was defined as the detection by microscopy of any parasite in a placental or cord blood smear.

The risk of peripheral malaria infection was analysed with a mixed-effects Poisson model [37,38]. Since the occurrence of malaria before enrolment in the study could not be observed (left censoring), the at-risk time period was defined as the interval from study enrolment to delivery. Lead time bias was (partially) accounted for by including the gestational age at enrolment as a covariate. Each individual follow-up (from enrollment to delivery) was split into intervals elapsing from one visit to another, and the log duration of these intervals was included as an offset. Baseline risk was modeled using a spline function. The level of parasitaemia (log transformed) was analysed using a linear model. When more than one malaria episode was observed in a pregnancy, the maximal parasitaemia level recorded per episode was used as a dependent variable. The presence of fever and the occurrence of placental malaria infection were analysed with logistic models. In each model, maternal age, gravidity, HIV status, education level, residency area (rural versus urban), and gestational age at inclusion were considered as potential risk factors.

The number of IPT doses was introduced as a time-dependent covariate in the model for peripheral malaria risk. However, IPT was interrupted after the treatment of a malaria infection, making the number of IPT doses an endogenous variable [39]. Since data were censored at the first malaria episode, only the relationship between the number of IPT doses received up to the beginning of a time interval and the risk of the first malaria episode during this time interval was assessed (using a log-linear model).

### Birth outcomes

The adverse outcomes evaluated in this study were stillbirth, preterm delivery, low birth weight and IUGR. IUGR was defined as a birth weight below the 10th percentile of the birth weight-for-gestational age. Type I (symmetric) IUGR and type II (asymmetric) IUGR were distinguished according to whether the Rohrer index was above the 10th percentile of Rohrer index for gestational age or not. United States population-based references were used as standard [40,41]. The association of each outcome with the various parameters of malaria exposure and with maternal characteristics were analysed separately for the full cohort, the subset of mother--newborn pairs with no or only one peripheral malaria infection, and the subset of mother--newborn pairs with ultrasound assessment of gestational age at baseline. Maternal age, education level (no education, primary level or ≥secondary level), residence area (rural versus urban), HIV status, number of clinic follow-up visits before birth outcome (<4 versus ≥4), and the newborn’s gender and gestational age at birth were included in all models. Stillbirth was analysed as a binary variable using a logistic model. Preterm delivery was analysed with gestational age at birth included as a continuous or binary variable (gestational age <37 weeks) using respectively a linear and logistic model. Weight and length at birth were both considered as continuous variables and analysed with a linear model adjusted for gestational age at birth. Parasitaemia was categorized as none, low (log parasitaemia ≤6 log parasites/μL) or high (>6 log parasites/μL). Late malaria infection was defined as a peripheral malaria infection occurring in the last two weeks before delivery. To better understand the effect of malaria infection timing independently of the enrolment timing, the association between birth weight and gestational age at infection (<15, 15-<20, 20-<24, ≥24 weeks) was analysed with a linear model restricted to the subset of mothers with no or only one malaria infection and with a gestational age <15 weeks at enrolment.

All analyses were performed using the open source statistical software R [42].

### Ethical approval

Written informed consent for study participation was obtained from all participants to the study. The study was approved by the institutional review boards of Mbarara University of Science and Technology, Uganda National Council for Science and Technology, and France’s “Comité de Protection des Personnes - Ile-de-France XI”. This study was registered with ClinicalTrials.gov, number NCT00495508.

## Results

### Study population characteristics

**Figure 1:**
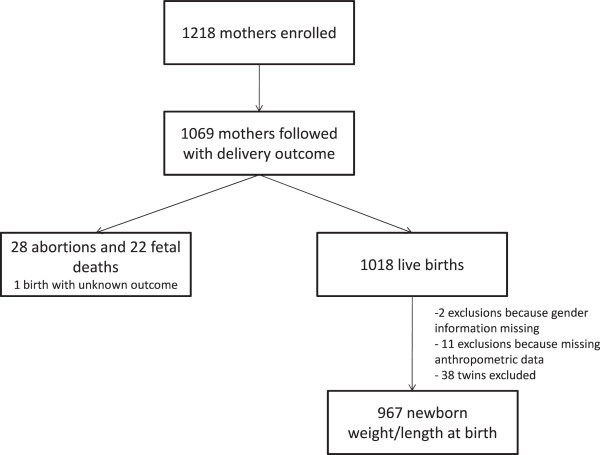
Flow chart.

Of the 1,218 women enrolled in the cohort, 149 (12%) were excluded from this analysis because they were lost to follow-up before their pregnancy reached an outcome (Figure 1). These excluded women were younger and had shorter follow-up (p=0.0001). One maternal death unrelated to malaria was observed (from sepsis five days after a caesarean section for obstructed labour in a term pregnancy).

Characteristics of the 1,069 women included in this cohort are summarized in Table 1. Women with malaria were enrolled somewhat later in their pregnancy than those with no infection detected (Table 1). Most of the mothers delivered in a health facility (84% at the regional hospital and 3% in a private clinic), with the remainder delivering at home. Median gestational age at enrolment was 19 weeks (Inter Quartile Range, IQR: 16–22) and median follow-up time was 21 weeks (IQR: 16–24). Almost 50% of the mothers reported the use of bed nets before the first visit. The mean number of visits, equivalent to the mean number of screening tests, was high (18, IQR: 10–23) (Table 1).

**Table 1:** Characteristics of the study population

		**No MiP**	**MiP**
**Mother characteristics at inclusion (n = 1069)**		**n=714**	**n=355**
Median age, year (IQR)		24 (21 – 27)	22 (19 – 25)
Median gestational age, week (IQR)		18 (15 – 20)	21 (17 – 28)
Residence, n (%)			
Urban		429 (60)	118 (33)
Rural		285 (40)	236 (67)
Place of delivery, n (%)			
Health facility		633 (88%)	291 (82%)
Home/transport		76 (11%)	62 (17%)
Education level, n (%)			
No education		68 (10)	47 (13)
Primary		285 (40)	210 (59)
≥ Secondary		361 (50)	98 (28)
HIV status, n (%)			
Negative		533 (74)	255 (71)
Positive		114 (16)	34 (10)
Unknown		67 (10)	66 (19)
Primigravid, n (%)		241 (34)	160 (45)
Delivery outcomes			
Abortion		23 (3)	5 (1)
Stillbirth		13 (2)	9 (2)
Mean number of visits (IQR)		20 (17 – 23)	16 (10 – 22)
**Newborn characteristics – liveborn singletons (n =967)**			
Gestational age at birth, weeks (IQR)		40 (39 – 41)	40 (39 – 41)
Preterm delivery, n (%)		42 (7)	29 (9)
Median weight, kg (IQR)		3.11 (2.87 – 3.44)	3.095 (2.80 – 3.33)
Median length, cm (IQR)		50 (47 – 51)	49 (48 – 51)
Female, n (%)		344 (54)	183 (56)

Mothers without ultrasound assessment of gestational age were more likely to live in remote rural areas (OR: 1.68, 95%CI: 1.21 - 2.31) and to be at a more advanced stage of the pregnancy at enrolment (mean gestational at enrolment was 23.5 weeks in mothers without ultrasound, versus 18.5 weeks in those with ultrasound, p<0.001). Of the 1,018 live births, 40 were excluded from the analysis on birth weight and length (Figure 1).

### Malaria exposure during pregnancy

#### Peripheral malaria infection

A total of 304 (28%) women had one or more malaria infections detected by peripheral blood smear (all species included) during follow-up visits, resulting in a total of 361 peripheral malaria infections (range: 1–4 malaria infections). Of the 242 (67%) infections recorded at inclusion, all involved *Plasmodium falciparum*, with six mixed infections. The 111 subsequent malaria infections included three mixed infections and 16 infections with non-falciparum species. Of the 55 positive RDT results with negative blood smear, 31 (52%) were observed at inclusion but no detectable microscopic parasitaemia were identified, while 24 (48%) were observed later during the follow-up period but with no previously documented infection. Peripheral malaria infection was associated with fever in only 16% of cases (n=62/361). The geometric mean (range) of parasitaemia was 1669 (24 – 302 500) parasites/μL. There were 23 women who had malaria and were not in the trial. Of them, 13 (57%) received quinine, 6 (26%) artemether-lumefantrine and the information was missing for 4 patients (17%).

In multivariate analysis, the risk of peripheral malaria was increased in mothers who were infected with HIV, were younger, primigravidae, had a lower education level, lived in rural areas and did not report bed net use at enrolment (Table 2, left column). In contrast, gestational age at inclusion was negatively associated with the risk of malaria infection. Compared to women who did not receive any IPTp, those who received one or two doses had an almost five or ten-fold reduction, respectively, in malaria infection risk (adjusted relative risk, RRa: 0.20, 95%CI: 0.14 - 0.30 and 0.10, 95%CI: 0.06 - 0.18). Women who experienced ≥2 malaria episodes were more likely to come from a rural area (2.23, 95%CI: 1.01 - 4.89) and to have enrolled in the study later during their pregnancy (OR: 0.95, 95%CI: 0.91 - 1.00) than those who had only one episode. Maximum parasitaemia was higher in women who were infected with HIV, included late during gestation or experienced recurrent malaria infections (Table 2, central column). Women with recurrent infection showed no significant difference in parasitaemia level between the first and subsequent infections (p=0.7).

**Table 2:** Risk factors for peripheral malaria during pregnancy, parasitaemia and placental malaria (multivariate analysis)

		**Peripheral malaria (rate ratio)**	**Parasitaemia (log)**	**Placental malaria (Odds ratio)**
Maternal age^a^		0.97 (0.94 - 0.98)	−0.01 (−0.04 to 0.01)	0.90 (0.80 - 1.02)
Education level				
Primary level		0.76 (0.56 - 1.04)	−0.10 (−0.40 to 0.20)	1.01 (0.17 - 5.81)
≥ secondary level		0.50 (0.35 - 0.72)	−0.22 (−0.57 to 0.12)	2.05 (0.33 - 12.64)
Rural residence		1.97 (1.54 - 2.52)	0.04 (−0.18 to 0.27)	6.00 (1.29 - 28.02)
Primigravidae		1.31 (1.02 - 1.69)	0.08 (−0.17 0.33)	1.00 (0.33 – 3.00)
Use of bednet		0.71 (0.56 - 0.90)	−0.08 (−0.30 to 0.14)	0.91 (0.30 - 2.77)
Gestational age at inclusion^b^		1.05 (1.03 - 1.07)	0.02 (0.00 to 0.04)	1.14 (1.05 - 1.24)
HIV status		0.92 (0.62 - 1.38)	0.42 (0.04 to 0.79)	2.68 (0.61 - 11.74)
# IPT doses				
1		0.25 (0.17 – 0.35)	‐‐	‐‐
2		0.10 (0.06 – 0.18)	‐‐	0.92 (0.22 – 3.76)
# malaria episodes				
1		‐‐‐	‐‐‐	3.30 (0.69 -15.88)
≥2		‐‐‐	0.47 (0.19 to 0.74)	15.80 (2.77 - 90.27)

^a^.By 10 years; ^b^. by week; IPT: intermittent preventive treatment.

#### Placental malaria infection

Of the 665 placental smears available, parasites were observed in 20 (3%) and pigment in 17 (2.5%) cases. The presence of pigment was associated with detectable parasites in 12 (7%) of the cases. Most of the infected placentas (17/20) came from mothers who had peripheral malaria detected during their enrolment in the study; in the remaining three women, no peripheral microscopic or positive RDT was detected during pregnancy or at delivery. Conversely, almost all of the placental biopsies from mothers without malaria detected during pregnancy had no haemozoin deposition nor parasitized erythrocytes by histology (72/79). All eight malaria infections in mothers with detectable parasites, but no pigment in the placental smear were observed during the third trimester. In adjusted analysis, the occurrence of a placental infection was associated with the number of peripheral malaria episodes, but not with parity (Table 2, right column). Similar results were seen in the subset of women with only one malaria infection (OR: 8.89, 95%CI: 1.07 - 74.21 for rural versus urban residency; 1.14, 95%CI: 1.03 - 1.27 for each additional week in gestational age, and 1.51, 95%CI: 1.03 - 2.22 for each log10 increase in parasitaemia level).

The association between the time since last malaria infection and the risk of placental malaria was assessed in a multivariate model restricted to women who experienced at least one malaria infection. Risk of placental malaria was negatively associated with the interval between the last peripheral infection and delivery (OR: 0.992, 95%CI: 0.985 - 0.998 per week) and positively associated with parasitaemia level (OR: 1.06, 95%CI: 1.02 -1.10 per log10 increase). No microscopic placental malaria was detected in women with a positive RDT, but negative blood smear.

### Delivery outcomes

#### Miscarriage and stillbirth

A total of 28 (3%) miscarriages and 22 (2%) stillbirths were observed in the study cohort. In adjusted analysis, the risk of miscarriage or stillbirth was significantly increased in mothers who were HIV-infected, living in urban areas or completed fewer follow-up visits (Table 3). Notably, in a model adjusted for HIV status and area of residence, malaria within two weeks of delivery was associated with a twofold greater risk of stillbirth (OR: 2.15, 95%CI: 1.04-4.46). It was not possible to include both the number of follow-up visits and the occurrence of malaria infection late during gestation as covariates in the same model because of their association with one another.

**Table 3:** Risk factors for adverse birth outcomes (multivariate analysis)

	**Stillbirth/ abortion**	**Preterm delivery (<37 wks)**	
		**Full dataset**	**Mother with US**
Urban residency	2.60 (1.31 - 5.16)	1.38 (0.81 - 2.34)	1.49 (0.79 - 2.83)
HIV status	2.70 (1.19 - 6.11)	2.33 (1.17 - 4.64)	3.21 (1.43 - 7.22)
# follow-up visits	0.74 (0.70 - 0.79)	0.86 (0.83 - 0.90)	0.81 (0.76 - 0.87)

#### Pre-term delivery

Overall 65 (7%) live-born pre-term deliveries were observed, with 45 (6%) occurring in the sub-group of mothers with ultrasound estimation of gestational age. In adjusted analysis of both the full cohort data and the subset with ultrasound, the risk of pre-term delivery was increased in women infected with HIV and in those with fewer follow-up visits (Table 3). As with stillbirth, an association between the risk of pre-term delivery and the occurrence of a malaria infection within the last two weeks of pregnancy was observed when the number of follow-up visits was dropped from the model, in both the full cohort dataset and the ultrasound subset (adjusted OR were 1.91, 95%CI: 1.05 – 3.50 and 2.84, 95%CI: 1.26 – 6.38, respectively).

#### Effect of malaria on gestation

**Figure 2:**
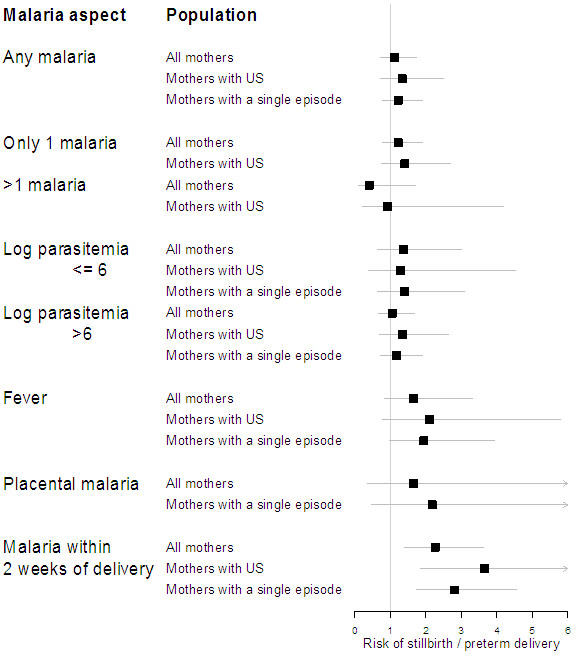
Summary of the association between risk of stillbirth or preterm delivery and different parameters of malaria exposure during pregnancy (odds ratio with 95% confidence intervals from analyses adjusted for maternal characteristics).

In adjusted analysis, shortened gestation (because of miscarriage, stillbirth or pre-term delivery) was associated with the occurrence of malaria infection within the last two weeks of pregnancy (Figure 2). A borderline association of shortened gestation with febrile malaria infection associated was also found (p=0.06).

### Weight and length at birth

**Figure 3:**
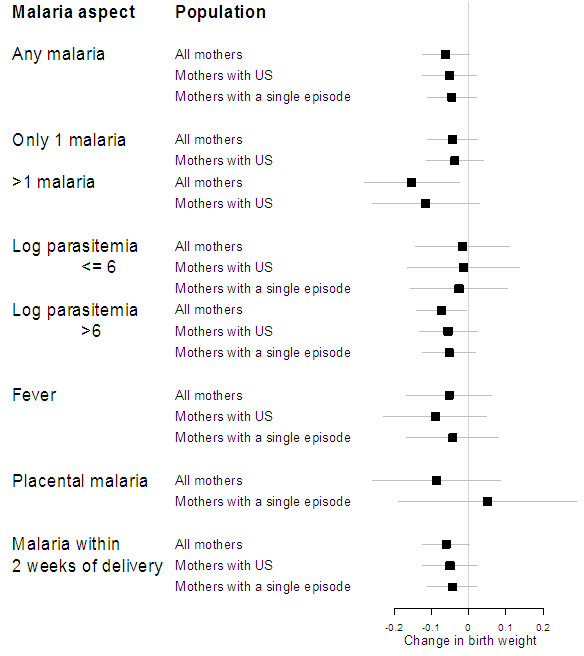
Summary of the association between birth weight and different parameters of malaria exposure during pregnancy (mean change with 95% confidence intervals from analyses adjusted for maternal characteristics).

In total, there were 57 (7%) infants with low birth weight, 39 (6%) with type II IUGR (asymmetric) and 89 (13%) type I IUGR (symmetric). In analysis adjusted for gender and for estimated gestational age at delivery, birth weight was reduced in primiparae, in mothers with low education level and in those who attended ≤4 follow-up visits (Table 4). Greater birth weight was observed in mothers with higher average hemoglobin level during pregnancy (+30g, 95%CI: 10–50), however this association disappeared in multivariate analyses adjusted for MiP. Of the various parameters of malaria exposure during pregnancy, all but placental malaria and symptomatic malaria were associated with lower weight at birth (Figure 3). However, the presence of malaria pigment in the placental smear was associated with reduced birth weight in univariate analysis (−0.26, 95%CI: -0.49 to −0.032) though not in adjusted analysis (p=0.2). Type I IUGR was not associated with any aspect of malaria in pregnancy (Additional file 2). By contrast, type II IUGR was increased in mothers with more than one malaria episode and with symptomatic malaria (Additional file 3). Median gestational age at last malaria infection was 21 weeks (19–22) in women with type I IUGR and 23 weeks (22–24) in women with type II IUGR (p=0.08).

**Table 4:** Factors associated with weight and length at birth (multivariate analysis, n = 967)

		**Weight at birth**	**Length at birth**
Newborn gender^a^		−0.01 (−0.07 to 0.04)	−0.03 (−0.44 to 0.38)
Maternal age^b^		0.05 (−0.02 to 0.12)	0.32 (−0.19 to 0.83)
Education level			
Primary level		0.06 (−0.03 to 0.15)	−0.25 -0.92 0.42
≥ Secondary level		0.10 (0.00 to 0.20)	−0.05 -0.75 0.65
Rural residency		−0.01 (−0.07 to 0.05)	−0.54 -0.97 -0.11
Primigravidae		−0.12 (−0.19 to −0.05)	−0.38 -0.88 0.11
HIV status		0.02 (−0.06 to 0.11)	−0.20 -0.82 0.41
>4 follow-up visits		0.45 (−0.01 to 0.92)	−2.42 -6.55 1.71
Gestational age at birth (wks)		0.11 (0.09 to 0.12)	0.67 (0.56 to 0.77)
Any peripheral malaria		−0.07 (−0.13 to 0.00)	0.17 (−0.30 to 0.64)

^a^ Female versus male; ^b^. by 10 years.

Analyses restricted to mothers with ultrasound-verified gestational age, or to those with no or only one malaria infection, found qualitatively similar associations, although sometimes with only borderline significance (Figure 3). Birth weight was not associated with the timing of the first malaria infection in mothers with gestational age <15 weeks at enrolment (p=0.8). This conclusion did not change when the cut-offs used for gestational age at enrolment was varied. No association was found between malaria exposure during pregnancy and newborn length.

## Discussion

The novel features of this cohort study are the frequent, intensive malaria screenings (median of 21 screens per pregnancy) and the provision of treatment based on the presence of parasite in the blood rather than on symptoms—practices which differ markedly from those common in endemic Africa. Another strength of this study is the accurate determination of gestational age for the majority of pregnancies.

Our results suggest that peripheral malaria infections during pregnancy, including those occurring late during gestation, contribute significantly to perinatal morbidity. Malaria infection at the end of pregnancy and those with fever rather than other aspects of malaria exposure, were associated more specifically with miscarriage or pre-term delivery. A similar association between malaria infections with fever and an increased risk of miscarriage has been reported in mothers with a single malaria episode during the first trimester of pregnancy [13]. Likewise, increased infant mortality has been reported after symptomatic malaria infections occurring at the end of the pregnancy [10,43]. In low endemic areas, 80% of microscopically detected infections become symptomatic if left untreated [44]. Since women in this cohort were treated if they had a positive blood smear, irrespective of whether they showed symptoms, it seems likely that this early detection and treatment of asymptomatic infections prevented higher rates of miscarriage and pre-term delivery. Current WHO policy calls for “the administration of at least two doses of SP during the second and third trimesters of pregnancy” [45,46]. More effective protection during late pregnancy is critical in low-endemic settings such as Mbarara, and addition of an extra (third) SP dose for all pregnant women rather than (as per current WHO policy) only to HIV-infected women, or monthly dosing, could provide more effective protection in all pregnancies.

Adjusting for gestational age at birth, we found that peripheral malaria infection during pregnancy was associated with lower birth weight, and that this association was consistently seen in both the full dataset and the subset of mother with ultrasound examination. Furthermore, more severe birth weight impairment was observed after multiple malaria infections and in malaria infections with high parasitaemia, even when IPT and bed net use was reported. These findings underscore the importance of implementing efficacious prevention, prompt diagnosis and highly effective anti-malarial treatment during pregnancy [47].

In addition to primigravidity, a well-known risk factor for MiP [1,4], it was found that low education level and rural residence were independently associated with malaria during pregnancy. These findings further support the notion that it is essential to scale up malaria prevention efforts in more isolated and deprived communities as recently highlighted in a meta-analysis of datasets from 25 African countries [48]. A low number of antenatal visits was also associated with reduced birth weight. The emphasis on at least four antenatal visits is required for improved control of malaria in pregnancy [45].

A limitation of this study was that documentation of malaria infection began only after the first trimester of pregnancy, resulting in left censored data. Women with multiple infections were more likely to have been enrolled later during their gestation and, therefore, early infections might have been missed. This could explain the absence of association in this analysis between MiP early during gestation and low birth weight, in contrast to results from other studies [11,14,15]. Alternatively, effective treatment of a single infection may allow recovery from infection and catch-up growth *in utero*.

Placental malaria has been shown to be a key intermediate factor in the pathological pathway of malaria [2,4,18,19]. However in our study, the proportion of placental infections (as determined from placental smears) was low, which most likely reflects our study’s intensive detection and rapid treatment of malaria infections, as previously observed [20]. However, for placental malaria diagnosis the sensitivity of parasite (rather than pigment) on placenta smear is low, so the actual proportion of placental infection might also have been underestimated [49]. In a cohort study of women actively screened and tested in the Gambia, the presence of pigment was reported to better reflect past infection with malaria [50], a finding which may explain why an association between low birth weight and the presence of pigment but not with the presence of parasite in placental smear was found in univariate analysis. Nevertheless, as the placenta cannot be examined until delivery, hence until after the adverse effect has already occurred, its utility for clinical diagnosis and prevention remains limited.

On the other hand, peripheral parasitaemia, which was associated with impaired delivery outcomes in this cohort, can be detected by frequent screening, so that prompt treatment can be given and adverse effects of the infection reduced. Since preventive efforts (IPT with SP and insecticide-treated bed net) still leave a large proportion of women with parasitaemia, taking the opportunity to screen women when they present to antenatal care is a strategy that should be considered. However, diagnostics for MiP remain problematic, since pregnant women often have low levels of parasitaemia and require diagnostic tools with greater sensitivity than microscopy (and good specificity)—for example, the Loop-mediated isothermal amplification (LAMP) [49,51]. As malaria prevalence decreases, the risk-to-benefit ratio for providing IPT also reduces. Hence efforts to determine the optimal number of screenings for women in malaria endemic areas are also required.

In conclusion, this study shows that the timing, parasitaemia, symptoms and number of peripherally detected malaria infections observed during pregnancy are associated with adverse outcomes. Prompt detection and treatment with an effective anti-malarial should be offered, irrespective of symptoms and use of other preventive measures in pregnancy. While frequent screening was associated with improved birth outcome, reaching mothers living in remote areas to prevent late attendance and low number of visits at antenatal care is essential, as they are more likely to suffer from poor outcomes.

## Competing interest

The authors declare that they have no competing interests.

## Authors’ contributions

PDB conducted the statistical analysis and wrote the paper. RM and PP designed the study, participated in the statistical analysis and manuscript drafting. ET participated in data collection, statistical analysis, and manuscript drafting. LW participated to the statistical analysis and manuscript review. CN and BT participated in the data collection, and manuscript review. YB participated in data collection, data analysis, and manuscript review. AM performed the histological analysis, interpreted the results and reviewed the manuscript. PG participated to the study design and manuscript review. All authors read and approved the final manuscript.

## Supplementary Material

Additional file 1 **Appendix.** Application of a multiple measures model using symphysis-pubis fundal height to predict gestational age in Ugandan pregnant women. Click here for file

Additional file 2 Summary of the association between type I intra uterine growth restriction and different parameters of malaria exposure during pregnancy (mean change with 95% confidence intervals from analyses adjusted for maternal characteristics). Click here for file

Additional file 3 Summary of the association between type II intra uterine growth restriction and different parameters of malaria exposure during pregnancy (mean change with 95% confidence intervals from analyses adjusted for maternal characteristics). Click here for file
