# Supplementary material for: Impact of malaria during pregnancy on pregnancy outcomes in a Ugandan prospective cohort with intensive malaria screening and prompt treatment
Source: Malar J. 2013 Apr 24;12:139. doi: 10.1186/1475-2875-12-139 (PMC3642015; doi:10.1186/1475-2875-12-139)
Supplement: Additional file 1 — Appendix. Application of a multiple measures model using symphysis-pubis fundal height to predict gestational age in Ugandan pregnant women. [file 1475-2875-12-139-S1.doc]

# Appendix - Application of a multiple measures model using symphysis-pubis fundal height to predict gestational age in Ugandan pregnant women

50% of mothers with both ultrasound measurements taken before 24 weeks and at least 3 symphysis-pubis fundal height (SFH) measurements taken on or before the malaria episode (if malaria) were randomly selected. The model (Model 3 in [34]) was applied to this data set and the parameters of the model were estimated using the same method.

The parameter estimates are as follows: cL0=3.9, cL1=0.83, c0=12, c1=0.7, c2=-0.03, c3=-0.3, k12=0.16, k13=6, k23=-0.5, Gmin=30 and Gmax=43.

The distribution of gestational age estimated using FH compares favorably with that estimated using ultrasound (histogram below).


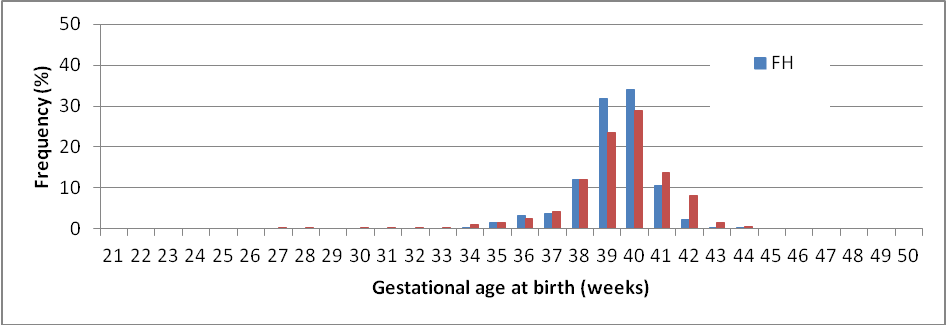


The residual error was ditributed evenly about zero (below).


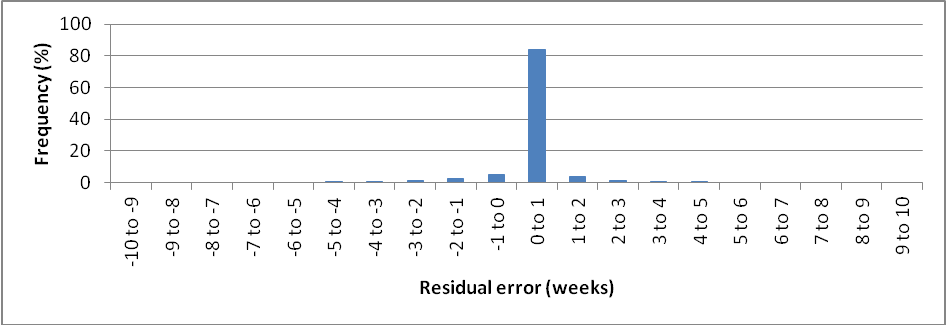


The model was then used to predict the remaining 50% of the data (below).


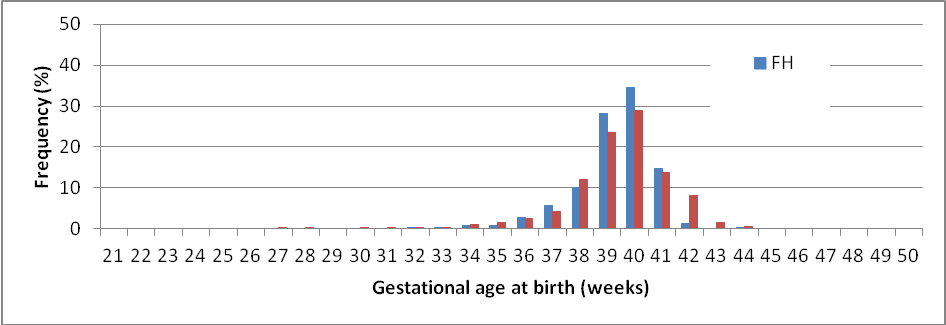


The residual error was ditributed evenly about zero (below).


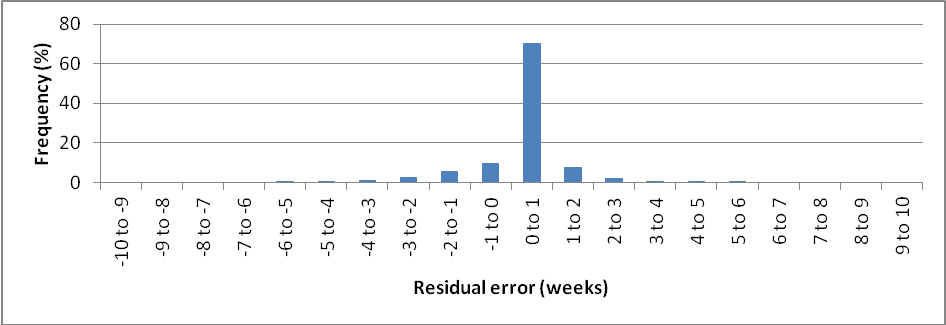


The model was then used to predict the EGA for patients with no ultrasound measurement with an accuracy of about ± 2 weeks (assuming a 90% confidence interval).
